# Evaluation of Monitored Anesthesia Care Involving Sedation and Axillary Nerve Block for Day-Case Hand Surgery

**DOI:** 10.3390/healthcare10020313

**Published:** 2022-02-07

**Authors:** Antoine Sanchez, Jan Chrusciel, Yann Cimino, Maxime Nguyen, Pierre-Grégoire Guinot, Stéphane Sanchez, Belaid Bouhemad

**Affiliations:** 1 Department of Anaesthesiology and Intensive Care, C.H.U. Dijon, 21709 Dijon, France; sanchez.antoine@live.fr (A.S.); maxime.nguyen-soenen@chu-dijon.fr (M.N.); guinotpierregregoire@gmail.com (P.-G.G.); 2 Department of Public Health and Performance, CH Troyes, 10003 Troyes, France; jan.chrusciel@ch-troyes.fr (J.C.); stephane.sanchez@ch-troyes.fr (S.S.); 3 Department of Anaesthesiology, Hôpital Privé Dijon—Bourgogne, 21000 Dijon, France; yanncimino@hotmail.com; 4 Lipides Nutrition Cancer—UMR 866 INSERM 1231, Université Bourgogne Franche-Comté, 21709 Dijon, France

**Keywords:** ABPB, intravenous sedation, hand surgery, ventilatory depression

## Abstract

Background: Ultrasound-guided axillary brachial plexus block (ABPB) is a technique of choice for regional anesthesia during hand and forearm surgery. Intravenous sedation may facilitate this procedure, particularly for those suffering from anxiety; however, it can also be associated with respiratory, cardiovascular, and neurological side effects. The objective of this study was to evaluate the effect of intravenous sedation on perioperative respiratory depression for patients undergoing day-case hand surgery under ABPB. Methods: A prospective, observational, single-center study was conducted between 1 May and 1 November 2016. Results: A total of 2318 patients were included, with 501 patients in the group with IV sedation and 1817 in the group without. A multivariable propensity-score matched analysis showed that the variables associated with the number of desaturation were: (i) sedation (aRR 1.534 [95% CI: 1.283 to 1.836]), (ii) age and sex, (iii) type of surgery, and iv) Body Mass Index (BMI). Conclusions: Supplementing ABPB with IV sedation was associated with an increased rate of respiratory depression (episodes of desaturation) compared to fully awakened patients. The rate of oxygen administration was also higher in sedated patients even though they had fewer cases of chronic respiratory diseases and fewer were active smokers than non-sedated patients. Future research should consider precisely evaluating patient satisfaction, as well as the differences between sedation and drug-free approaches.

## 1. Introduction

Monitored Anesthesia Care (MAC) during hand and forearm surgery usually involves Axillary Brachial Plexus Block (ABPB) to control pain and ensure that a limb is immobile as well as conscious during sedation to diminish patient anxiety concerns [1,2]. Current recommendations advise the use of ultrasound-guided ABPB since it increases the effectiveness of the block and decreases the rate of complications [3,4]. However, even with ultrasound guidance and conscious sedation, ABPB may still be difficult to perform and may be uncomfortable for the patient, particularly if they suffer from anxiety disorders [5]. Some drug-free approaches, such as music, can be used in the operating theater, which have been shown to be effective in decreasing perioperative pain [6,7]. 

Intravenous (IV) conscious sedation reduces anxiety more effectively [8]; however, the drugs used for this type of sedation can cause respiratory, cardiovascular, and neurological side effects [9]. Recommendations from the Society of Anesthesia and Sleep Medicine state that caution must be exercised regarding the use of sedative drugs, especially with benzodiazepines and in patients with obstructive sleep apnea, obesity, cirrhosis, chronic kidney disease, cardiovascular diseases, and with the elderly [10,11,12]. These recommendations were recently complemented in a report by the American Society of Anesthesiologists [13]. This report focused on procedural sedation and analgesia, where observation and monitoring of patients in an appropriately staffed and equipped environment were encouraged after sedation/analgesia until they reached their baseline level of consciousness and were no longer at increased risk for cardiorespiratory depression. However, in some cases, the use of a sedation procedure has been incompatible with day-case surgery since it increases the time spent in the post-anesthesia care unit due to respiratory issues [14].

The primary aim of this study was to evaluate the effect of conscious IV sedation on perioperative ventilatory depression in patients undergoing day-case hand surgery under ABPB. The secondary aims were to study the effect of conscious IV sedation on perioperative hemodynamic parameters, patient satisfaction, and average time spent in the post-anesthesia care unit (PACU) and determine the variables associated with a risk of desaturation that could influence an anesthesiologists’ decision to use sedation.

## 2. Methods

### 2.1. Study Design and Population

A prospective observational study evaluating usual ABPB procedures at the Fontaine-Lès-Dijon Clinic between 1 May 2016 and 1 November 2016 was carried out. Patients undergoing day-case hand surgery under ABPB were eligible for this study. The inclusion criteria were age ≥18 years, American Society of Anesthesiologists (ASA) physical status score of 1 to 3, and were undergoing day-case hand surgery (either planned or emergency) under ultrasound-guided ABPB. The patient’s consent was recorded orally.

Patients with hand surgery were referred to our center, and therefore there were no patients with other trauma. The types of surgeries performed in our study were mainly: carpal tunnel, dupuytren, trapezio-metacarpal prosthesis, jump fingers, and De Quervain’s tenosynovectomy. Most emergency surgeries involved fractures or hand injuries. 

The exclusion criteria were refusal to participate, patients <18 years of age, pregnancy, major dementia that would prevent employing usual procedures or data collection, and chronic respiratory failure. The sample was a convenience sample based on the maximum number of patients available during the study period. 

Blocks were conducted in a dedicated block room. Patients were then transferred to the operating room and then to the PACU. The ultrasound machine used to perform the ABPB was a NextGen LOGIQ by General Electric with a linear, 47mm, high frequency 4–13Mhz probe. The patients were placed in a supine position; their arm was abducted 90° from the body at the shoulder and flexed 90° at the elbow. After appropriate skin preparation with 2% chlorhexidine plus alcohol, the block was performed. The probe was placed in the transverse plane at the lateral border of the pectoralis major muscle to obtain the best view of the brachial plexus. The practicians used an in-plane approach with a short-beveled, 5-cm, 22G insulated needle; after that, each nerve was identified and blocked separately, and an ultrasound-guided block of the medial brachial cutaneous and the intercostal nerves was performed to prevent pain associated with the use of tourniquets [15].

### 2.2. Data Collection

Patients admitted for planned surgery received either oral premedication with alprazolam one hour before being transferred to the operating theater or no premedication in accord with the arrangement chosen during the pre-anesthesia consultation. Patients admitted for emergency surgery were transferred directly to the operating theater with no premedication. 

Patients were compared according to the use of IV sedation. During regional anesthesia, the attending physician was free to use IV sedation in accordance with the patient’s wishes, by either administering midazolam (1 to 2 milligrams depending on the patient’s weight and level of anxiety) or by an association of drugs (1 to 2 milligrams of midazolam associated with propofol (20 mg maximum), sufentanil (5 ug maximum), or ketamine (10 mg maximum)). The choice of local anesthetic and/or administration of oxygen was left to the attending physician. The level of anxiety was a subjective evaluation by the physician, and no objective criteria were used to decide if IV sedation drugs should be used. The ABPB was ultrasound-guided, and music could also be provided with headphones for a patient to listen to. The choice between mepivacaine and ropivacaine was left to the discretion of the anesthesiologist who performed the block. Most of the time, mepivacaine was used for short, painless, post-operative surgeries, such as carpal tunnel. Ropivacaine was used for long surgery that caused postoperative pain, such as fractures.

Patients were then transferred to the operating room for surgery, after which they were monitored in the PACU following the usual criteria for postoperative observation. Patient satisfaction was rated on a ten-point Likert scale. On the day following the procedure, patients were contacted by telephone and asked about any post-surgery complications that may have occurred. 

Data collected included: age, gender, ASA score, height, weight, history of arterial hypertension, chronic respiratory disease (asthma, COPD, sleep apnea, and emphysema), smoking habits, antihypertensive medication or beta-blockers, body mass index (BMI), type of surgery (emergency or planned), attending surgeon and anesthesiologist’s names, operation site, premedication and the molecule used, sedation methods and the molecule used, presence of music during the procedure, choice of local anesthetic and adjuvant (if applicable), oxygen administration, mean systolic and diastolic blood pressure in the operating theater, mean oxygen saturation in the operating theater, mean heart rate in the operating theater, number of episodes of decreased oxygen saturation in the operating theater, pain in the PACU (visual analogue scale), a patients Aldrete score when leaving the PACU, patient satisfaction in the PACU, time spent in the operating room, time spent in the PACU, and the total time spent on site. 

Data collected by telephone the following day included: whether the call was answered or not, and if so, if there was any pain during the night after surgery, pain medication taken, nausea and/or vomiting, food intake, presence of fever, bleeding, rate of compliance with prescribed treatment(s), and any presence of dyssomnia. 

### 2.3. Primary and Secondary Outcomes

The primary outcome was the number of oxygen desaturation, which was defined as pulse oximetry readings (SpO2) below 93% (measured by digital pulse oximetry) and which required physician intervention. Monitoring of SpO2 began upon arrival in the regional anesthesia preparation room and continued until departure from the PACU. In case of desaturation associated with somnolence, verbal stimulation was conducted. In case of persistent desaturation, oxygen was provided at 4 L/min with a nasal cannula. Secondary outcomes were oxygen administration, total time spent in the operating theater, patient satisfaction with the monitored anesthesia care evaluated in the recovery room, mean heart rate, and systolic and diastolic blood pressure in the operating theater. 

### 2.4. Statistical Methods

Qualitative variables were presented as absolute frequencies with associated percentages. Normally distributed quantitative variables were presented as means with their standard deviations (SDs). Quantitative variables that were not normally distributed were presented as medians with the interquartile range. Qualitative variables were compared between sedated patients and non-sedated patients using a Chi-squared test or a Fisher’s exact test according to their distribution. Normally distributed quantitative variables were compared using a Student’s *t*-test, and non-normal quantitative variables were compared using Mann–Whitney’s U test.

### 2.5. Propensity Score Analysis

To account for the non-randomized nature of the data, a propensity score analysis was conducted. We estimated the predicted probability of sedation using a multivariable logistic regression model. The variables included in the model were those associated with a risk of desaturation that could influence the anesthesiologists’ decision to use sedation. These variables were: respiratory disease, age, sex, BMI, anesthesiologist’s name, operating surgeon’s name, and the ASA score. To reduce indication bias, we matched patients by using their calculated propensity scores. A fixed ratio of 1:1 matching without replacement was also used. Patients who could not be matched to a similar patient in the opposite group were excluded from the analysis to limit indication bias.

A multivariable analysis was also performed as an additional step to account for residual confounding that could be present after matching. This analysis was based on a negative binomial (NB) model with the number of desaturation episodes as the independent variable. The NB model was used because of the right-skewed nature of the outcome, which has a higher variance than would be expected with a Poisson distribution. The main parameter of interest in our study was the exponentiated regression coefficient of the sedation variable.

The variables included in the multivariable model were sedation, ASA score, age (included as a continuous variable), gender, time in the operating room (included as a continuous variable), type of surgery (emergency or other), respiratory disease, BMI (included as a continuous variable), high blood pressure (yes/no), and any use of a beta-blocker. Data management and matching were realized using R version 4.0.0 (www.R-project.org). Multivariable analysis was performed using SAS version 9.3 (SAS Institute Inc., Cary, NC, USA).

### 2.6. Legal Requirements

In accordance with the law on personal data protection (N°78-17, 6 January 1978), our study was submitted to the National Commission of Data Protection (CNIL) via the hospital’s personal data protection correspondent (Declaration N°1975811v0) on 16 July 2016. Patients received an informational leaflet concerning the design and aims of the study, their right to refuse to participate, and their right to leave it at any time (Appendix 1). Their consent was recorded orally. ABPB and anesthesia case studies are common practices in our center. The data used in the study were also routinely collected during such procedures. 

## 3. Results

**Figure 1:**
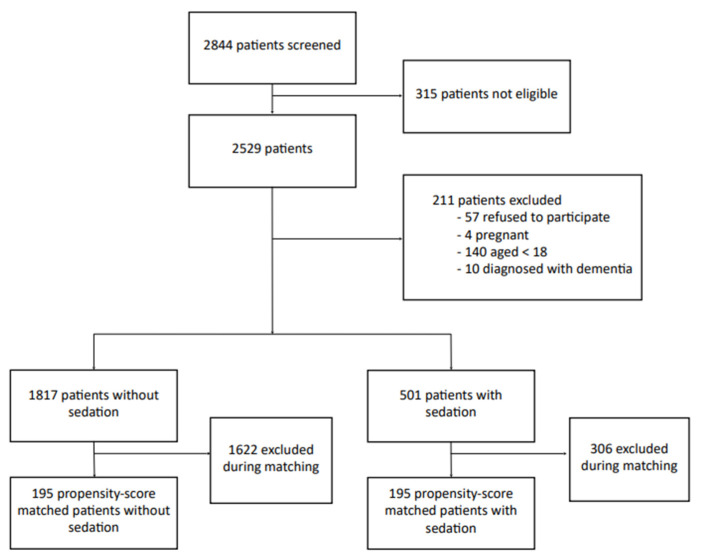
Flow chart of the study to assess monitored anesthesia care for day-case hand surgery.

During the study inclusion period, 2844 patients were screened (Figure 1). Of these, 315 patients were not eligible, either because they were hospitalized or they were operated on several times during this period. A total of 211 patients were excluded: 57 refused to participate, 4 were pregnant, 140 were <18 years of age, and 10 had dementia. A total of 2318 patients were, therefore, included, with 501 in the sedated group and 1817 in the non-sedated group. The characteristics of both groups are listed in Table 1. The frequency of chronic respiratory disease (10% vs. 6%, *p* = 0.009) and active smoking was lower in the sedated group (7% vs. 13%, *p* < 0.0001). Sedation more frequently involved the use of midazolam alone rather than an association of sedative drugs (83% of single-drug sedation, 17% multiple-drug sedation).

The median number of desaturation was higher in the sedated group than in the non-sedated group: 3 (Q1–Q3 1–17) vs. 1 (Q1–Q3 0–7; *p* = 0.002). The highest number of desaturation was recorded in patients sedated with multiple drugs: median 3.5 (Q1–Q3 1–33). The administration of oxygen was more frequent in sedated patients (5% vs. 0.3%, *p* < 0.0001). In addition, scores of patient satisfaction were lower (60.2 % of maximum scores vs. 67.0%). The median time spent in the recovery room was longer in the sedated group (38 min vs. 30 min, *p* < 0.0001), as was the median time spent in the operating room (97 min vs. 95 min, although the difference was not statistically significant: *p* = 0.26). Information collected by phone on the following day is presented in Table 2. Of the 1477 patients contacted, 652 replied. Sedated patients had more pain the day after surgery (23% vs. 13%, *p* = 0.006). There were no between-group differences related to pain during the first night, use of pain medication, nausea/vomiting, food intake, fever, bleeding, treatment compliance, or dyssomnia. 

### 3.1. Propensity-Score Based Matching

**Figure 2:**
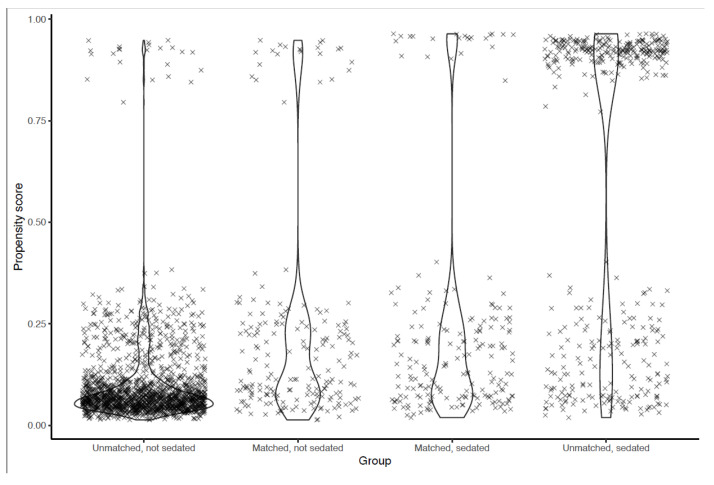
Distribution of propensity scores before and after matching.

**Figure 3:**
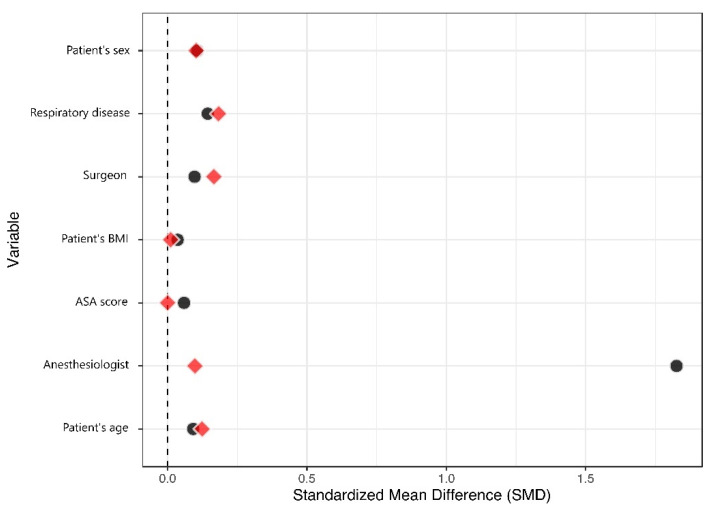
Love plot.

One hundred and ninety-five sedated patients were able to be matched to a similar control (Figure 2). The matched dataset, therefore, contained 390 patients (Table 3). The primary improvement regarding balance was for the anesthesiologist variable, with a change in the standardized mean difference (SMD) from 1.83 to 0.10, at a comparatively lower cost regarding the balance of other variables (Figure 3). All SMDs were <0.20 after matching, indicating that the covariate balance was acceptable. Residual confounding was further accounted for in the multivariable analysis.

Data points represent individual patients. A high propensity score means that the patient’s characteristics make them more likely to receive the treatment. Propensity scores in the matched population are presented side by side at the center. The maximum difference in propensity scores for a matched pair was 0.057.

Standardized Mean Differences (SMD) for the covariates used in the propensity score calculation is presented before and after matching. A high SMD indicates that the treatment group is different from the control group for the relevant covariate. SMDs in the matched population are shown in red. After matching on the propensity score, SMDs for all covariates were <0.20, indicating an acceptable balance.

### 3.2. Multivariable Analysis

The dispersion coefficient of the multivariable model was 2.57 [95% CI 2.20 to 3.01], which suggests that the use of the NB model was adequate. Sedation was associated with a higher number of desaturation (aRR 1.534 [95% CI 1.283 to 1.836]) (Table 4). Other variables associated with the number of desaturation episodes were gender (male), procedure as emergency surgery, older age, and higher BMI.

## 4. Discussion

The results of this study showed that when ABPB is associated with sedation, the rate of ventilatory depression (oxygen desaturation number) was higher than for ABPB alone. Furthermore, the rate of oxygen administration was higher for sedated patients even though the patients in this group had fewer chronic respiratory diseases and fewer were smokers. The time spent in the recovery room was also higher for sedated patients. The time spent in the operating room was not significantly associated with sedation; therefore, it is unlikely to explain our results. On the other hand, as patients are released from PACU when the effects of anesthesia and sedation wear out, any additional time spent in PACU can be seen, therefore, rather than a cause of the number of desaturation. Therefore, we did not adjust for time spent in PACU because such an adjustment could bias the results by masking a true effect of sedation. Lastly, patients who were sedated were less satisfied.

Limited information is available in the literature about the complications associated with IV sedation. This may mostly be because of the diverse types of MAC and the lack of clearly defined study protocols. An epidemiological study into the complications associated with sedation by the American Society of Anesthesiology published in 2006 [16] showed that most of the reported side effects were of respiratory nature and the result of excessive sedation; 75% of the patients who experienced respiratory side effects received an association of sedative drugs. However, insufficient information was available regarding the total number of anesthetized patients, thus it was impossible to evaluate the incidence of these side effects. In 2015, a Cochrane systematic review of sedation administered in emergency departments was unable to draw a conclusion regarding the effectiveness and safety of the different types of existing sedation due to the low number of sufficient quality clinical trials available [17]. 

In this study, the rate of desaturation increased with sedation. In addition, oxygen was administered to 5% of sedated patients compared to only 0.3% of non-sedated patients. The rate of hemodynamic side effects, however, was not higher in the sedated group, which was probably due to the high proportion of patients sedated by midazolam, which has fewer hemodynamic effects than other sedative drugs [18]. 

Satisfaction scores were lower in the sedated group even though they were generally very high in our study. The clinical significance of this difference is therefore limited. The high satisfaction scores may not be surprising since patients who undergo day-case surgery are usually very satisfied with their care [19]. They also tend to be less anxious than patients undergoing major surgery [20].

An important secondary finding was the increase in the time spent on-site, which was over ten minutes longer in the sedated group and was mainly due to the increased time spent in the recovery room. This increase, which could be attributed to the neurological effects of midazolam, is consistent with previous findings [21]. Since the 2018-934 regulations on post-surgical monitoring were published on 29 October 2018, it is possible that patients who undergo surgery with regional anesthesia and not with IV sedation avoid the recovery room altogether [22]. Furthermore, longer recovery-room time has been shown to be a source of patient dissatisfaction [23]. This must be considered when determining the risk-benefit of administering sedation since it requires a systematic stay in the recovery room. 

Only 28% of patients were successfully contacted on the day following their surgery. This response rate is lower than previous studies that had a contact rate between 35% and 52% [24]. Our findings, therefore, must be interpreted with caution. The low rate of complications, such as nausea, bleeding and fever, is consistent with the standard complication rate for this type of surgery [25]. There was a significant difference between the two groups concerning pain on the first day after surgery, which may be related to the greater use of short-duration local anesthetics in this group. However, this result may be affected by selection bias because of the low response rate.

### Strengths and Limits

To the best of our knowledge, this is the first study to evaluate the effects of sedation during regional anesthesia in an operating theater in a large cohort of sedated and non-sedated patients. 

Our study had several limitations. First, it was open-label, single-center, and non-randomized. The results are therefore subject to selection and interpretation biases since the choice of sedation was left to the discretion of the attending physician. However, the groups were generally comparable except for the presence of respiratory disease. This may suggest that anesthesiologists were more reluctant to sedate patients with a history of respiratory problems. It would be reasonable to expect that this choice would have led to a lower rate of ventilatory depression in the sedated group; however, the results show the opposite and suggest that in spite of the methodological limitations of this study, there is a tangible effect of sedation on respiratory function.

Measurement error may have occurred for pulse oximetry recordings because SpO2 and blood pressure were measured on the same limb, given that the other limb was positioned within the surgical field. It has been reported that measuring blood pressure can decrease SpO2 [26]; however, since both groups were monitored in the same way, the difference in the mean number of desaturation suggests a direct effect from sedation. 

The pertinence of patient satisfaction measurement using a simple Likert-type numeric scale may be questionable given the complexity of evaluating such a variable [27]. A multi-modal regional anesthesia satisfaction scale, such as the EVAN-LR scale [28], could have been more appropriate; however, the large number of questions involved made it impractical for it to be used in our department. Additionally, we did not objectively evaluate patient anxiety using scores, which has been shown to be related to satisfaction [29]. Patients with anxiety disorders could, therefore, be a confounder in the interpretation of the results related to satisfaction. The number of desaturation was highest in patients sedated with multiple drugs; however, our multivariable analysis did not distinguish between patients based on the number of drugs they received. Further studies should explore the differences between sedation by midazolam and sedation with multiple drugs.

## 5. Conclusions

The results found in this study suggest that sedation increases perioperative ventilatory depression without increasing patient satisfaction. Thus, the use of sedation may not comply with current rapid rehabilitation objectives during day-case hand surgery. Further studies are needed to accurately evaluate patient satisfaction, whether different drugs used for sedation would yield different outcomes, and the differences between sedation and non-sedation techniques.

## Figures and Tables

**Table 1:** Baseline characteristics according to the study groups of monitored anesthesia care for day-case hand surgery.

Characteristics	Sedated Group	Non-Sedated Group	*p*-Value	Unadjusted OR [95%CI]	Total
**Number of patients**	501 (22%)	1817 (78%)			2318 (100%)
Sex			0.077	0.835 [0.684 to 1.019]	
*Male*	276 (55%)	1081 (60%)			1357 (59%)
*Female*	225 (45%)	736 (40%)			961 (41%)
**Age (years), mean (SD)**	49 (17)	50 (17)	0.052		
**Height (cm), mean (SD)**	169 (9)	170 (9)	0.232		
**Weight (kg), mean (SD)**	75 (17)	76 (16)	0.269		
**BMI (kg/m^2^), mean (SD)**	26 (5)	26 (5)	0.504		
**ASA**			0.285		
*1*	307 (61%)	1038 (57%)			1345 (58%)
*2*	162 (33%)	638 (35%)			800 (35%)
*3*	31 (6%)	137 (8%)			168 (7%)
**Hypertension**			0.129	0.827 [0.647 to 1.057]	
*Yes*	99 (20%)	417 (23%)			516 (22%)
*No*	402 (80%)	1400 (77%)			1802 (78%)
**Anti-hypertensive medication**			0.099	0.809 [0.630 to 1.040]	
*Yes*	93 (19%)	403 (22%)			496 (21%)
*No*	408 (81%)	1413 (78%)			1821 (79%)
**Beta-blockers**			0.19	0.781 [0.539 to 1.131]	
*Yes*	37 (7%)	170 (9%)			207 (9%)
*No*	464 (93%)	1646 (91%)			2110 (91%)
**Chronic respiratory disease**			0.009	0.579 [0.388 to 0.864]	
*Yes*	30 (6%)	180 (10%)			210 (9%)
*No*	471 (94%)	1637 (90%)			2108 (91%)
**Smoking**			<0.0001	0.511 [0.355 to 0.737]	
*Yes*	36 (7%)	239 (13%)			275 (12%)
*No*	465 (93%)	1582 (87%)			2043 (88%)
**Reason for hospitalization**			0.451	1.079 [0.885 to 1.316]	
*Emergency surgery*	249 (50%)	858 (47%)			1107 (48%)
*Planned surgery*	252 (50%)	957 (53%)			1209 (52%)
**Operation site**			0.64	1.047 [0.859 to 1.278]	
*Right*	250 (50%)	926 (51%)			1176 (51%)
*Left*	250 (50%)	884 (49%)			1134 (49%)
**Local anesthetic**			0.056	1.221 [0.994 to 1.501]	
*Mepivacaine*	322 (64%)	1077 (59%)			1399 (60%)
*Ropivacaine*	179 (36%)	735 (41%)			914 (40%)
**Premedication**			<0.0001	1.658 [1.317 to 2.088]	
*Yes*	136 (28%)	337 (19%)			473 (20%)
*No*	357 (72%)	1467 (81%)			1824 (80%)
**Music**			0.003	0.649 [0.490 to 0.861]	
*Yes*	67 (13%)	349 (19%)			416 (18%)
*No*	434 (87%)	1468 (81%)			1902 (82%)
**Type of sedation**					
*Midazolam*	418 (83%)	0 (0%)			418 (83%)
*Multiple*	83 (17%)	0 (0%)			83 (17%)
**Oxygen**			<0.0001	17.193 [7.058 to 41.882]	
*Yes*	27 (5%)	6 (0.3%)			33 (1%)
*No*	474 (95%)	1811 (99.7%)			2285 (99%)
**Mean systolic BP (mmHg), mean (SD)**	130 (17)	133 (16)	0.001		
**Mean diastolic BP (mmHg), mean (SD)**	78 (10)	78 (10)	0.571		
**Mean pulse oximetry (%), mean (SD)**	96 (2)	96 (2)	0.189		
**Number of desaturations, median (Q1–Q3)**	3 (1 to 17)	1 (0 to 7)	<0.0001		
**Mean heart rate (bpm), mean (SD)**	72 (12)	70 (11)	0.019		
**Satisfaction in recovery room**			0.049		
10	210 (60.2%)	914 (67.0%)			1127 (65.7%)
9	93 (26.6%)	309 (22.7%)			402 (23.4%)
≤8	46 (13.2%)	140 (10.3%)			187 (10.9%)
**Time in operating room (minutes), median (Q1–Q3)**	97 (74 to 129)	95 (69 to 128)	0.26		
**Time in recovery room (minutes), median (Q1–Q3)**	38 (25 to 56)	30 (18 to 47)	<0.0001		
**Total time spent on site (minutes), median (Q1–Q3)**	148 (120 to 189)	138 (108 to 177)	<0.0001		

Note: Data provided are numbers (%) or means (SD).

**Table 2:** Day 1 follow-up call according to the sedated and non-sedated study groups of monitored anesthesia care for day-case hand surgery.

Day 1 Follow-Up	Sedated Group	Non-Sedated Group	*p*-Value	Unadjusted OR [95%CI]	Total
**Answered call on day 1**			0.405	0.902 [0.707 to 1.150]	
*Yes*	146 (29%)	506 (28%)			652 (28%)
*No*	200 (40%)	625 (34%)			825 (36%)
**Pain on day 1**			0.006	2.005 [1.222 to 3.291]	
*Yes*	29 (23%)	59 (13%)			88 (15%)
*No*	100 (77%)	408 (87%)			508 (85%)
**Pain the first night**			0.205	1.308 [0.863 to 1.983]	
*Yes*	44 (34%)	131 (28%)			175 (29%)
*No*	86 (66%)	335 (72%)			421 (71%)
**Pain medication taken**			0.065	1.477 [0.976 to 2.233]	
*Yes*	107 (74%)	328 (66%)			435 (67%)
*No*	38 (26%)	172 (34%)			210 (33%)
**Nausea/Vomiting**			0.799	1.161 [0.369 to 3.655]	
*Yes*	4 (3%)	12 (2%)			16 (2%)
*No*	141 (97%)	491 (98%)			632 (98%)
**Food intake**			0.157	1.624 [0.829 to 3.179]	
*Yes*	135 (93%)	446 (88%)			581 (89%)
*No*	11(7%)	59 (12%)			70 (11%)
**Fever**			0.113	7 [0.630 to 77.750]	
*Yes*	2 (1%)	1 (0.2%)			3 (1%)
*No*	144 (99%)	504 (99.8%)			648 (99%)
**Bleeding**			0.692	1.396 [0.268 to 7.270]	
*Yes*	2 (1%)	5 (1%)			7 (1%)
*No*	143 (99%)	499 (99%)			642 (99%)
**Treatment compliance**			0.055	1.872 [0.896 to 3.553]	
*Yes*	132 (92%)	429 (86%)			561 (87%)
*No*	12 (8%)	73 (134%)			85 (13%)
**Dyssomnia**			0.775	1.162 [0.415 to 3.253]	
*Yes*	5 (3%)	15 (3%)			20 (3%)
*No*	140 (97%)	488 (97%)			628 (97%)

Note: Data provided are numbers (%). Patients with a VAS ≤ 3 were considered as having no pain.

**Table 3:** Baseline characteristics in the matched dataset after matching for confounders associated with sedation of patients with monitored anesthesia care for day-case hand surgery.

Variable	No Sedation	Sedation	*P*-Value after Matching	Standardized Mean Difference (SMD) after Matching	Standardized Mean Difference (SMD) before Matching
**n**	195	195			
**Anesthetist, n (%)**			0.96	0.10	1.83
*Anesthetist 1*	23 (11.8)	32 (16.4)			
*Anesthetist 2*	41 (21.0)	39 (20.0)			
*Anesthetist 3*	38 (19.5)	40 (20.5)			
*Anesthetist 4*	31 (15.9)	32 (16.4)			
*Anesthetist 5*	30 (15.4)	23 (11.8)			
*Anesthetist 6*	32 (16.4)	29 (14.9)			
**Surgeon, n (%)**			0.75	0.17	0.10
*Surgeon 1*	23 (11.8)	23 (11.8)			
*Surgeon 2*	9 (4.6)	6 (3.1)			
*Surgeon 3*	24 (12.3)	24 (12.3)			
*Surgeon 4*	32 (16.4)	29 (14.9)			
*Surgeon 5*	75 (38.5)	78 (40.0)			
*Surgeon 6*	32 (16.4)	35 (17.9)			
**Respiratory disease, n (%)**	18 (9.2)	9 (4.6)	0.07	0.18	0.14
**Age: mean (SD)**	49.95 ± 18.06	47.82 ± 16.23	0.22	0.12	0.09
**Sex (male), n (%)**	96 (49.2)	86 (44.1)	0.31	0.10	0.11
**ASA score, n (%)**			0.99	0.00	0.06
*ASA1*	117 (60.0)	117 (60.0)			
*ASA2*	65 (33.3)	65 (33.3)			
*ASA3*	13 (6.7)	13 (6.7)			
**BMI, mean (SD)**	25.70 ± 5.02	25.64 ± 4.96	0.91	0.01	0.04

SD: Standard Deviation, Data provided are numbers (%) or means (SD).

**Table 4:** Multivariable analysis by negative binomial (NB) regression modelling of the number of desaturations in the propensity-score matched dataset to assess monitored anesthesia care for day-case hand surgery.

Variable	RR [95%CI]	*p*-Value (Univariate Analysis)	aRR [95%CI]	*p*-Value (Multivariable Analysis)
**Sedation**	1.383 [1.141 to 1.678]	<0.0001	1.534 [1.283 to 1.836]	<0.0001
**ASA score**		<0.0001		0.97
*ASA 1*	1 [Reference]		1 [Reference]	
*ASA 2*	1.238 [0.873 to 1.718]		0.992 [0.714 to 1.362]	
*ASA 3*	1.657 [1.050 to 2.908]		1.051 [0.670 to 1.763]	
**Age (1 unit increase)**	1.055 [1.043 to 1.067]	<0.0001	1.045 [1.031 to 1.059]	<0.0001
**Sex: Male (Reference = female)**	1.103 [0.908 to 1.342]	0.32	1.292 [1.076 to 1.551]	0.01
**Time in operating room**	1.002 [0.998 to 1.006]	0.45	1.003 [0.999 to 1.007]	0.12
**Emergency surgery**	0.768 [0.634 to 0.961]	0.002	0.765 [0.622 to 0.939]	0.01
**Respiratory disease**	0.991 [0.663 to 1.480]	0.37	0.937 [0.632 to 1.451]	0.76
**BMI (1 unit increase)**	1.126 [1.079 to 1.175]	<0.0001	1.086 [1.046 to 1.129]	<0.0001
**High blood pressure**	1.879 [1.523 to 2.363]	<0.001	1.108 [0.798 to 1.555]	0.54
**Beta blocker**	1.685 [1.243 to 2.283]	0.02	0.879 [0.608 to 1.288]	0.50
**Anesthetist**		0.93		0.12
*Anesthetist 1*	1 [Reference]		1 [Reference]	
*Anesthetist 2*	0.913 [0.425 to 2.474]		0.601 [0.294 to 1.417]	
*Anesthetist 3*	0.968 [0.593 to 1.677]		0.879 [0.570 to 1.409]	
*Anesthetist 4*	0.995 [0.632 to 1.632]		0.786 [0.509 to 1.240]	
*Anesthetist 5*	1.096 [0.768 to 1.561]		1.310 [0.935 to 1.832]	
*Anesthetist 6*	1.243 [0.800 to 1.999]		1.043 [0.696 to 1.593]	
**Surgeon**		0.52		0.57
*Surgeon 1*	1 [Reference]		1 [Reference]	
*Surgeon 2*	0.930 [0.633 to 1.416]		1.123 [0.784 to 1.645]	
*Surgeon 3*	1.219 [0.828 to 1.864]		1.336 [0.913 to 1.993]	
*Surgeon 4*	0.878 [0.578 to 1.401]		0.823 [0.557 to 1.251]	
*Surgeon 5*	0.719 [0.461 to 1.194]		0.993 [0.647 to 1.576]	
*Surgeon 6*	1.013 [0.664 to 1.627]		1.045 [0.676 to 1.663]	

*RR; Risk Ratio, aRR; adjusted Risk Ratio, CI; confidence interval.*

## Data Availability

The data are available upon request to the corresponding author belaid_bouhemad@hotmail.com.

## Abbreviations

**ABPB**	Axillary Brachial Plexus Block
**ASA**	American Society of Anesthesiologists
**BMI**	Body Mass Index
**IV**	Intravenous
**MAC**	Monitored Anesthesia Care
**NB**	Negative Binomial
**PACU**	Post-anesthesia Care Unit
